# Deep neural networks for genomic prediction do not estimate marker effects

**DOI:** 10.1002/tpg2.20147

**Published:** 2021-10-01

**Authors:** Jordan Ubbens, Isobel Parkin, Christina Eynck, Ian Stavness, Andrew G. Sharpe

**Affiliations:** ^1^ Global Institute for Food Security (GIFS) University of Saskatchewan Saskatoon SK S7N 0W9 Canada; ^2^ Agriculture and Agri‐Food Canada Saskatoon SK S7N 0X2 Canada; ^3^ Department of Computer Science University of Saskatchewan Saskatoon SK S7N 0W9 Canada

## Abstract

Genomic prediction is a promising technology for advancing both plant and animal breeding, with many different prediction models evaluated in the literature. It has been suggested that the ability of powerful nonlinear models, such as deep neural networks, to capture complex epistatic effects between markers offers advantages for genomic prediction. However, these methods tend not to outperform classical linear methods, leaving it an open question why this capacity to model nonlinear effects does not seem to result in better predictive capability. In this work, we propose the theory that, because of a previously described principle called shortcut learning, deep neural networks tend to base their predictions on overall genetic relatedness rather than on the effects of particular markers such as epistatic effects. Using several datasets of crop plants [lentil (*Lens culinaris* Medik.), wheat (*Triticum aestivum* L.), and *Brassica carinata* A. Braun], we demonstrate the network's indifference to the values of the markers by showing that the same network, provided with only the locations of matches between markers for two individuals, is able to perform prediction to the same level of accuracy.

## Core Ideas

- The capacity of deep neural networks does not match performance in genomic prediction.
- Deep neural networks are not disadvantaged when they cannot access values of the markers.
- Deep neural networks likely attend primarily to genetic relatedness, not marker effects.

**Abbreviations**
CNN: convolutional neural network
FC: fully connected neural network
GBLUP: genomic best linear unbiased predictor
GP: genomic prediction
QTN: quantitative trait nucleotide
SNP: single nucleotide polymorphism

## 1 INTRODUCTION

Genomic prediction (GP) is the practice of predicting trait values, such as yield, for members of a population using marker data. In the breeding of crop plants, GP has been shown to be an effective way to speed the rate of improvement for agronomically relevant traits (Crossa et al., 2010; Goddard & Hayes, 2007; Goddard et al., 2009). Genomic prediction has become increasingly accessible in recent times because of rapid improvements in genotyping technologies using next‐generation sequencing at a much‐reduced cost and has been integrated into many breeding programs around the world (Crossa et al., 2017).

The literature on GP in plants and animals is mature, with linear additive models of genetic effects being the most popular in practice. However, recent years have seen an increasing interest in using machine‐learning methods, including deep learning, for the GP task. Although it is widely assumed that deep neural networks will learn to model complex nonlinear epistatic effects (Ma et al., 2018), they rarely perform better than simple linear models such as the genomic best linear unbiased predictor (GBLUP) (Montesinos‐Lopez et al., 2021). This implies that deep neural networks either do not estimate the effects of specific alleles or do not benefit from doing so.

In this work, we explore the gap between the capacity and the real‐world performance of deep‐learning methods for GP by controlling what information the network is allowed to access. We develop a novel technique for training a deep neural network that performs prediction based on the locations of matches between markers for pairs of individuals without giving the network access to the marker data directly. Under the assumption that a deep neural network estimates marker effects, this training strategy is expected to perform poorly as the network has no information about which alleles are present in any individual. However, through experiments with different plant species and population structures, we show that this prediction strategy, based solely on genetic relatedness, performs just as well as allowing the network to access the values of the markers. The equivalence in predictive performance for deep neural networks with or without access to the marker data implies that these networks tend to rely on estimating overall genetic relatedness, not the effects of specific markers.

## 2 BACKGROUND

### 2.1 Kernel methods

The term kernel method refers to a class of models that rely on a fixed or learnable kernel function *K*, which relates a notion of similarity between its two inputs. Kernel machines are a common example of kernel methods in machine learning. In the simplest terms, kernel machines are models in the following form:

(1) $$y=g\left(\sum_{i}\;a_{i}y_{i}K\left(x,x_{i}\right)+b\right)$$

where *x* and *y* are the input and output of the model respectively, (*x_i_,y_i_*)*, i* ∈ {0*…N*} are the *N* samples of the training set (the data vectors and the labels), *g* is an optional transfer function, and *a_i_, i* ∈ {0*…N*} and *b* are learnable weight and bias terms. In this way, the output of the kernel machine for an input is a function of the weighted similarity between it and the training samples, as indicated by the kernel function *K*. Kernel functions have classically been chosen based on expert knowledge of the data or the problem space, for example, using Gaussian or polynomial kernels.

**Core Ideas**

- The capacity of deep neural networks does not match performance in genomic prediction.
- Deep neural networks are not disadvantaged when they cannot access values of the markers.
- Deep neural networks likely attend primarily to genetic relatedness, not marker effects.

In GP, the GBLUP is a common estimator of the following form:

(2) $$\text{BLUP}\left(\hat{g}\right)=\left[I+{\left(XX^{T}\right)}^{-1}\frac{\sigma_{\varepsilon }^{2}}{\sigma_{g}^{2}}\right]y$$

where **X** is the (mean‐centered and normalized) matrix of markers, and *σ*_ε_ and *σ*_g_ are the standard deviation of errors and genetic effects, respectively (Morota et al., 2014). Here, the covariance matrix **XX^T^** represents a simple linear kernel, capturing linear additive effects among markers. Nonlinear kernels can also be used in the estimation of genetic relatedness. Reproducing kernel Hilbert spaces regression with a Gaussian kernel has seen some success in the literature, although linear kernels still remain the most popular (Crossa et al., 2017).

### 2.2 Neural networks and deep learning

Nonlinear models, such as neural networks, have the theoretical capability to capture arbitrarily complex epistatic effects in whole‐genome regression. For example, multiple markers may have negligible effects individually but significant effects when considered in combination. The ability to model these interactions gives nonlinear models a potential representational advantage over linear models for prediction. Several authors have suggested that this advantage makes deep neural networks promising candidates for performing GP (Abdollahi‐Arpanahi et al., 2020; Ma et al., 2018; Ramstein et al., 2019).

Machine‐learning methods for GP, including deep‐learning methods, have been explored in the literature. The results have been mixed, with the performance of deep learning varying between datasets. One of the most commonly used neural network architectures is the convolutional neural network (CNN), which uses layers of filter banks to capture local patterns in the data. Despite their representational power and their success in other subfields of biology, CNNs have been shown to perform similarly to classical linear models in some cases, although somewhat underperforming them in others (Montesinos‐Lopez et al., 2021). One extensive survey of GP methods for various traits in six different plant species showed that CNNs performed the worst among all tested models, on average (Azodi et al., 2019). Another study in soybean [*Glycine max* (L.) Merr.] showed similar performance between deep‐learning and linear models when missing single nucleotide polymorphisms (SNPs) were imputed (Liu et al., 2019). Results for blueberry (*Dianella nigra* Colenso) and strawberry (*Fragaria* ×*ananassa* Duchesne ex Rozier) again saw no advantage for CNNs over conventional methods for most traits (Zingaretti et al., 2020).

The similarity in performance between powerful, high‐capacity, deep‐learning models and standard linear models suggests that either the genetic architectures of all the examined traits are strongly linear additive in nature or that deep neural networks are not estimating nonlinear effects between markers despite their ability to do so.

## 3 METHODS AND MATERIALS

### 3.1 Obscuring marker data from the network

Because of the black‐box nature of deep neural networks, it is difficult to determine precisely how the network arrives at a prediction (Wang et al., 2020). Therefore, the most reliable way to determine whether the network benefits from estimating the effects of particular alleles is to completely disallow the network from accessing the values of the markers themselves.

**FIGURE 1:**
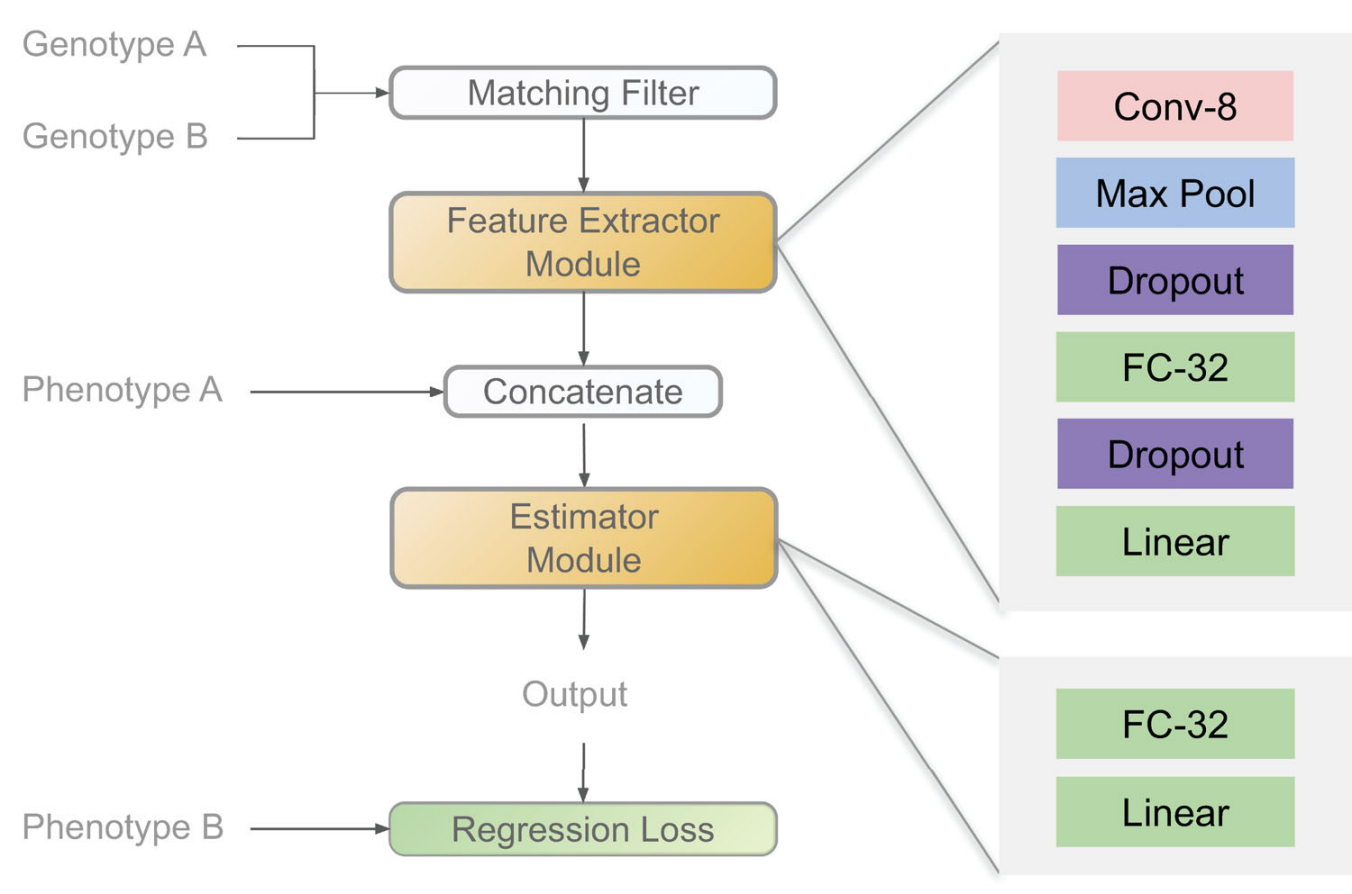
Overview of the proposed method, referred to as the obscured model, for training a deep neural network without giving the network access to the marker data. Inset, the DeepGS architecture (Ma et al., 2018) is shown as the feature extractor module, and a two‐layer fully connected network is shown as the estimator module

To investigate whether deep neural networks estimate phenotype based on estimating marker effects, we propose a novel training method that obscures the marker data from the network. A schematic of the obscured model is shown in Figure 1. Comparing with the kernel machine formulated in Equation 1, the obscured model can be represented as follows:

(3) $$y=E\left(f_{\mathrm{est}}\left\{f_{\mathrm{emb}}\left[\phi \left(x,x_{i}\right)\right],y_{i}\right\}\right)$$

where φ is the matching filter operation:

(4) $$\phi \left(u,v\right)=\left\{\begin{array}{c} 1,\text{if}\;u_{j}=v_{j}\\ 0,\text{otherwise} \end{array}\right.$$

for each marker *j*, *f*_emb_ is the feature extractor module and *f*_est_ is the estimator module. The matching filter only outputs one when the encodings match exactly, so comparing heterozygous and homozygous individuals will produce a zero. Here, the subscripted (*x_i_,y_i_*) is a member of the training population, called *the reference sample*, and (*x,y*) is the sample *x* for which the phenotype *y* is being predicted, called the query sample. In a manner similar to kernel machines, the final point estimate produced for a query sample is an aggregate of predictions made over all members of the training population.

During the training phase, the feature extractor module and the estimator module are trained in an end‐to‐end fashion using random pairs of samples from the training population. The training samples are retained for inference time, during which a forward pass is performed on each query sample using each member of the training population as the reference sample. Because the output is a distribution over predictions, the point estimate is given by the mean of this output distribution. Various other aggregation methods, such as ensemble learning and mixture‐of‐experts models (Jacobs et al., 1991), were trialed, with no consistent improvement over using the simple mean. In practical terms, any nonlinear models can be used for the feature extractor module and for the estimator module. Here, we use a simple two‐layer, feed‐forward neural network for the estimator, and we use the DeepGS CNN architecture from the literature for the feature extractor module (Ma et al., 2018). DeepGS is a small convolutional architecture, using a single convolutional layer and two fully connected layers. It uses stochastic regularization in the form of Dropout (Srivastava et al., 2014), which aims to mitigate overfitting by randomly zeroing units in the network. DeepGS was selected, as it is the most commonly used example of a deep network built specifically for GP. We use DeepGS here as this allows us to make direct comparisons to the DeepGS architecture used in a whole‐genome regression approach, as differences in performance are therefore not a result of differences in architecture or model capacity.

The obscured method is distinct from reproducing kernel Hilbert spaces regression methods in the fact that Equation 3 is not a reproducing kernel *K* (it is not guaranteed that *K*: *X* × *X* → R is positive semidefinite). It is also distinct from standard whole‐genome regression methods, as the network never has access to the marker data. It is only provided with the locations of matches between two otherwise anonymous samples, and the specific alleles of both samples are unknown. This means that the network cannot reduce to existing whole‐genome regression methods by regressing on the markers of the query sample while ignoring the reference sample.

Neural networks have previously been evaluated for GP using the pedigree relationships or the genomic relationship matrix as the input data (Gianola et al., 2011). This represents an extreme reduction in the amount of information available to the network, from the number of markers to the number of individuals in the training population and their respective linear relationships. In contrast, under the training methodology proposed here, the neural network retains the original input size and still has access to the full range of locality information present in the original signal. That is, unlike Gianola et al. (2011), the only information lost is the values of the alleles—the network is still free to find nonlinear relationships between any locations in the high‐dimensional input data (which now only provides information about relatedness).

### 3.2 Models evaluated

To test our hypothesis, we compare the obscured model, with DeepGS as the feature extractor, with DeepGS used in the typical fashion performing whole‐genome regression with direct access to the marker data. For completeness, we also compare these networks with two common linear models, GBLUP, LASSO, as well as two other machine‐learning methods: a fully connected neural network (FC, and a fully connected neural network with *L*_0_ regularization (Louizos et al., 2018).

LASSO is a penalized linear model of the following form:

(5) $$\text{argmi}n_{w}\;{\left(y-w^{T}x\right)}^{2}+\lambda {\left|w\right|}_{1}$$

where *w* are the model parameters and λ is a tunable constant that controls the strength of the regularization term. In practice, the *L*_1_ penalty on the weight vector tends to encourage sparsity in the weights, a desirable quality under the assumption that a low proportion of the total markers have influence on the trait. LASSO and a related model, the Elastic Net, performed the best (and better than the commonly used RR‐BLUP) in a survey of penalized linear models for GP (Ogutu et al., 2012).

Fully connected neural networks, also known in some GP literature as multilayer perceptrons, are a common choice for machine‐learning applications in GP. We use a standard two‐layer neural network. Batch normalization is used, as it potentially improves generalization performance and allows training to succeed over a higher range of learning rates.

Motivated by the principle of sparsity, we also investigate a fully‐connected neural network equipped with *L*_0_ regularization (Louizos et al., 2018). While *L*_1_ regularization penalizes the absolute magnitude of the weights, *L*_0_ regularization penalizes the proportion of nonzero weights, explicitly encouraging sparsity. Optimizing this regularization term using gradient descent methods is impossible because the *L*_0_ norm is not differentiable. However, it can be approximated through a hard concrete distribution—a continuous relaxation of discrete random variables (Jang et al., 2017; Maddison et al., 2019). The result is differentiable feature selection learned simultaneously with the model (Abid et al., 2019).

### 3.3 Hyperparameter selection

In order to fairly represent the performance of each method, care must be taken to tune them individually for optimum performance. In the machine‐learning literature, constants used in the model specification or during training, such as the learning rate or the size in units of a hidden layer, are referred to as hyperparameters. The process of selecting the values for hyperparameters that are likely to result in the best performance on the test data is known as hyperparameter tuning. This is done by holding out a portion of the training data called the validation set. Performance on the validation set can be used as a proxy for performance on the unseen test data, allowing an automated search over values of the hyperparameters in either a random or a structured fashion. In this section we describe how hyperparameters were selected for each of the six methods.

For the LASSO model, the regularization coefficient *λ* = 0.001 is used, as this value allows convergence in every dataset. Tuning *λ* resulted in slightly worse performance overall, and the bounds for tuning must be set carefully or values for *λ* may be selected that cause nonconvergence when training the final model. For the FC and *L*_0_ models, the number of hidden units and the learning rate are determined via hyperparameter search as this consistently improved performance over predetermined constants. We use an exponentially decaying temperature schedule for the *L*_0_ model (Abid et al., 2019). This schedule for the temperature parameter defines how the variable selection changes from a continuous output to a discrete output over the course of training. For the DeepGS network, most hyperparameters are prescribed by the authors of the DeepGS architecture. We use a learning rate of 1 × 10^−5^. We found that tuning the learning rate does not improve performance and sometimes results in learning rates that are infeasible for training the final model.

For the obscured model, hyperparameters were predetermined, as hyperparameter tuning proved to be both costly and the performance gains inconsistent. We use a value of *d* = 1 for the dimensionality of the embedding emitted by the feature extractor module. Smaller values of *d* tend to be better for small datasets and so we find this to be the most general. The number of hidden units in the estimator module is set to 32, as tuning did not change performance significantly.

In addition to hyperparameter tuning, the validation set can also be used for early stopping. Some models may see a decrease in error on the validation set until a certain number of training steps and increasing error thereafter. Halting training at this inflection point can prevent the model from overfitting the training data. However, in all experiments presented here, we found that it was universally better to use all of the training data to train the model instead of reserving a portion as a validation set to monitor for early stopping. This is consistent with previous reports that the size of the training population is one of the most important factors in predictive performance (Abdollahi‐Arpanahi et al., 2020). For all models, we add the validation set back into the training data after hyperparameter tuning (when applied) and use the entirety to train the final model. Each model was trained until the training error had ceased to decrease.

### 3.4 Datasets

To evaluate the difference between deep neural networks trained with and without access to the raw marker values, we use four datasets of food crops representing a variety of species and sample sizes. Sources for the data are provided in the final section.

The first two datasets we use for evaluation are published datasets of lentil (Haile et al., 2020). The authors of this study examined GP performance within‐population, across‐populations, and across‐environments using a variety of models. The authors published a lentil diversity panel made up of 320 individuals as well as two biparental populations termed LR‐01 and LR‐11. To evaluate the largest and most genetically diverse population possible, we use the combined LR‐01 and LR‐11 dataset from the paper constituting a total of 230 individuals. This data is an amalgamation of two different biparental populations, and the authors of the data reported lower predictive performance on average using the two populations combined as opposed to using each individually. This makes an interesting case study, given the unusual population structure in this challenging combined population. The 39,000 SNPs sampled via exome capture provided by the authors are used.

We also use a population of 2,536 *B. carinata* plants. This population is a nested association mapping population consisting of recombinant inbred line progeny from 50 crosses of different *B. carinata* lines with a common reference line. This dataset includes 17,000 SNPs.

For the final crop dataset, we use a published dataset of 2,403 landrace accessions of Iranian wheat (Crossa et al., 2016). The individuals were phenotyped for a variety of agronomically relevant traits and the authors of the dataset used these for a GP study with standard models. This is the dataset previously reported in the DeepGS paper, and so we include it in order to be consistent with the analysis from that publication (Ma et al., 2018). The dataset includes a set of 33,000 SNPs obtained via genotyping‐by‐sequencing.

Each dataset was split into five folds, and each model was trained five times using each fold as the hold‐out set. Hyperparameter tuning was performed within each split when applicable using one of the four training folds as the validation set.

3.5

**FIGURE 2:**
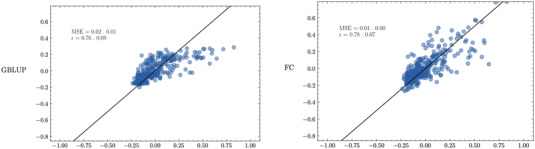
Example of scatter plots for the lentil diversity panel dataset using genomic best linear unbiased predictor (GBLUP) (left) and fully connected neural network (FC) (right). Although *r* is similar (0.76 and 0.78), the shape is different

As is usual in GP, we report Pearson's *r* between the predicted and actual phenotypes as the measure of predictive performance. A property of *r* that should be mentioned is the distribution of *r* values for two independent random variables. For samples drawn from two independent Gaussian distributions, the probability density for *r* is given by the following equation:

(6) $$f\left(x\right)=\frac{{\left(1-x^{2}\right)}^{\frac{N-4}{2}}}{B\left(0.5,\frac{N-2}{2}\right)}$$

where *B* is the beta function and *N* is the sample size. Clearly, for small values of *N*, the expected variance in *r* values is high. We see this difference in variance between the biparental lentil dataset, which is the smallest in sample size, and the *B. carinata* and wheat datasets, which are significantly larger. In practice, we found *r* values to be volatile, varying considerably between different random splits of the data, and different random initializations. Even multiple runs with identical random seeds produce slightly different results for the machine‐learning models based on nondeterministic effects alone. For this reason, we decline to describe a method as superior when the difference in *r* is small, taking sample size into account. Because of the volatile and opaque nature of *r* as an estimate of predictive performance (for example, Figure 2), we also provide scatter plots of the results as Supplemental Figures.

## 4 RESULTS

**FIGURE 3:**
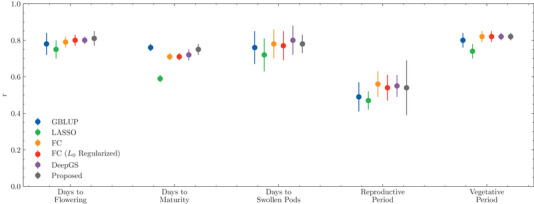
Genomic prediction results for the lentil diversity panel. Additional details are shown in Supplemental Figure S1

**FIGURE 4:**
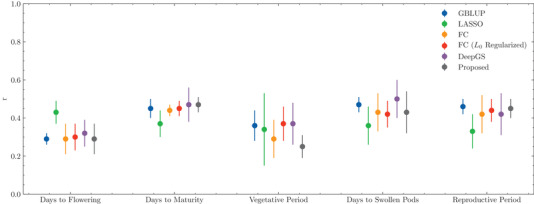
Genomic prediction results for lentil biparental populations. Additional details are shown in Supplemental Figure S2

**FIGURE 5:**
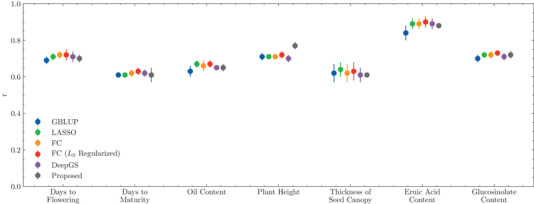
Genomic prediction results for *B. carinata*. Additional details are shown in Supplemental Figure S3

**FIGURE 6:**
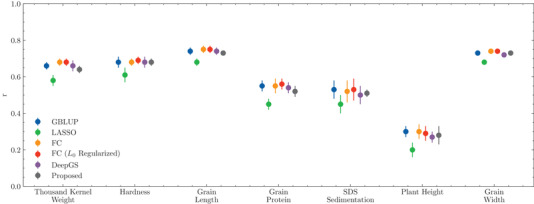
Genomic prediction results for wheat. Additional details are shown in Supplemental Figure S4

Prediction results for the lentil diversity panel, lentil biparental population, *B. carinata*, and wheat datasets are shown in Figure 3, Figure 4, Figure 5, and Figure 6, respectively.

For all experiments, all six methods seem to provide similar predictive performance, with no method consistently outside the margin of error. The exception is LASSO, for which we observe a small decrease in performance on the lentil diversity panel and wheat datasets relative to the other methods. LASSO also exhibits more volatile performance than the other methods across the experiments, likely a result of the difficulties of training penalized linear models and their dependence on the strength of the regularization term. Among all the methods investigated in our evaluations, GBLUP continues to prove itself as a flexible choice when the underlying genetic structure of the trait is unknown. Although it is only capable of modeling linear additive effects with constant variance, GBLUP showed strong performance across species and traits. Despite this relatively good predictive performance, it remains the simplest method to implement, requires no tuning, and terminates in considerably less time than any of the machine‐learning methods.

Overall, our results largely mirror the findings elsewhere in the literature—for sufficiently large datasets, most methods offer comparable predictive performance regardless of differences in model capacity or complexity. Most importantly, the obscured method did not perform significantly worse than the standard DeepGS model on any dataset even though it had no access to the marker data.

## 5 DISCUSSION

### 5.1 On shortcut learning

It is reasonable to assume that, having the capacity to model complex epistatic effects between markers, deep neural networks would use this capability during training. However, deep neural networks are known to rely on features of the input that happen to correlate with the cost function but that are not the features to which the network was meant to attend. This phenomenon has been termed ‘shortcut learning’^1^ (Geirhos et al., 2020). For example, it has been shown that neural networks attend to color and texture more than the form of objects when performing image classification. Learning these simpler, higher‐level features, or shortcuts, provides the network with a path of least resistance during optimization. The network does not need to learn a complicated representation of ‘cow’ if the presence of cows is correlated with green grass in the image—looking for green pixels in the lower half of the image will suffice (Geirhos et al., 2020).

The shortcut learning principle may be true of genomic data as well. The network may find it unnecessary to seek out a small number of quantitative trait nucleotides (QTNs), which are likely to be associated with a small and noisy gradient signal, when high‐performing individuals are likely to share large haplotype blocks in common by virtue of being genetically related. From the perspective of shortcut learning, it is not unlikely that the indifference of the network to the markers is due to the fact that it considers high‐level genetic relatedness as a viable shortcut for predicting the phenotype.

### 5.2 Limitations

One study reported that for simulated nonadditive genetic architectures, neural networks (FC networks and CNNs) performed slightly better than GBLUP for a 100‐QTN simulated trait, albeit slightly worse for a 1000‐QTN simulated trait (Abdollahi‐Arpanahi et al., 2020). This could be taken as evidence to suggest that there may be some nonlinear genetic architectures in which deep neural networks are advantaged because of their ability to estimate nonlinear effects. However, it is worth noting that for practically all real‐world datasets [in this study and in Abdollahi‐Arpanahi et al. (2020), as well as elsewhere in the literature (Azodi et al., 2019; Liu et al., 2019; Montesinos‐Lopez et al., 2021; Zingaretti et al., 2020)], this difference between neural networks and linear models is not observed. This discrepancy may be explained by differences in marker‐effect sizes between real and simulated traits or how biologically plausible the architecture of the simulated trait was in practice. These issues could have an effect on the behavior of the network in terms of which features are salient for prediction. Finally, these small performance differences could also be accounted for by, as we have observed, volatility in Pearson's *r*.

## 6 CONCLUSION

Compared with linear models, deep neural networks have the ability to model complex nonlinear effects; however, this capability does not seem to benefit them in GP. In this paper, we show that one can obscure the marker data from the network, blinding it to which alleles are present in each individual; however, doing this does not seem to be at all deleterious to performance. Taken together, these two observations offer evidence that deep neural networks generally do not learn to model epistatic effects at all but rather depend on estimating overall genetic relatedness. We suggest that this is consistent with the behavior of neural networks in other domains and is explained by a concept called shortcut learning.

This work reveals a clear direction for future work in deep learning for GP. The development of models that are capable of avoiding the learning of shortcuts in genomic data may unlock some of the potential of these powerful nonlinear models in genomic prediction. Unfortunately, the literature on shortcut learning is still nascent and there are currently no generally accepted solutions. However, this is an active area of research and including genomic data as part of the push forward on this problem will serve to benefit genomic applications of deep learning in the future.

## AUTHOR CONTRIBUTIONS

Jordan Ubbens: Conceptualization; Formal analysis; Investigation; Methodology; Software; Validation; Visualization; Writing‐original draft; Writing‐review & editing. Isobel Parkin: Resources; Writing‐review & editing. Christina Eynck: Resources; Writing‐review & editing. Ian Stavness: Funding acquisition; Resources; Supervision; Writing‐review & editing. Andrew Sharpe: Funding acquisition; Resources; Supervision; Writing‐review & editing.

## CONFLICT OF INTEREST

The authors declare no conflicts of interest.

## Supporting information

Supplemental Figure S1: Detailed results for the first three traits in the lentil diversity panel dataset. The line y = x is shown in black.
Supplemental Figure S2: Detailed results for the first three traits in the lentil biparental dataset. The line y = x is shown in black.
Supplemental Figure S3: Detailed results for the first three traits in the carinata dataset. The line y = x is shown in black.
Supplemental Figure S4: Detailed results for the first three traits in the wheat dataset. The line y = x is shown in black.

## Data Availability

The data used in the wheat and lentil experiments are available from the sources referenced by their authors (Crossa et al., 2016; Haile et al., 2020). The carinata data is available on request. Code for reproducing the method is available at https://www.github.com/p2irc/obscured.

## Footnotes

1 This is distinct from the use of the term *shortcut* to refer to skip connections in some neural network architectures.
