# Supplementary material for: Deep neural networks for genomic prediction do not estimate marker effects
Source: Plant Genome. 2021 Oct 1;14(3):e20147. doi: 10.1002/tpg2.20147 (PMC12807099; doi:10.1002/tpg2.20147)
Supplement: Supplementary file 1 [file TPG2-14-e20147-s001.pdf]

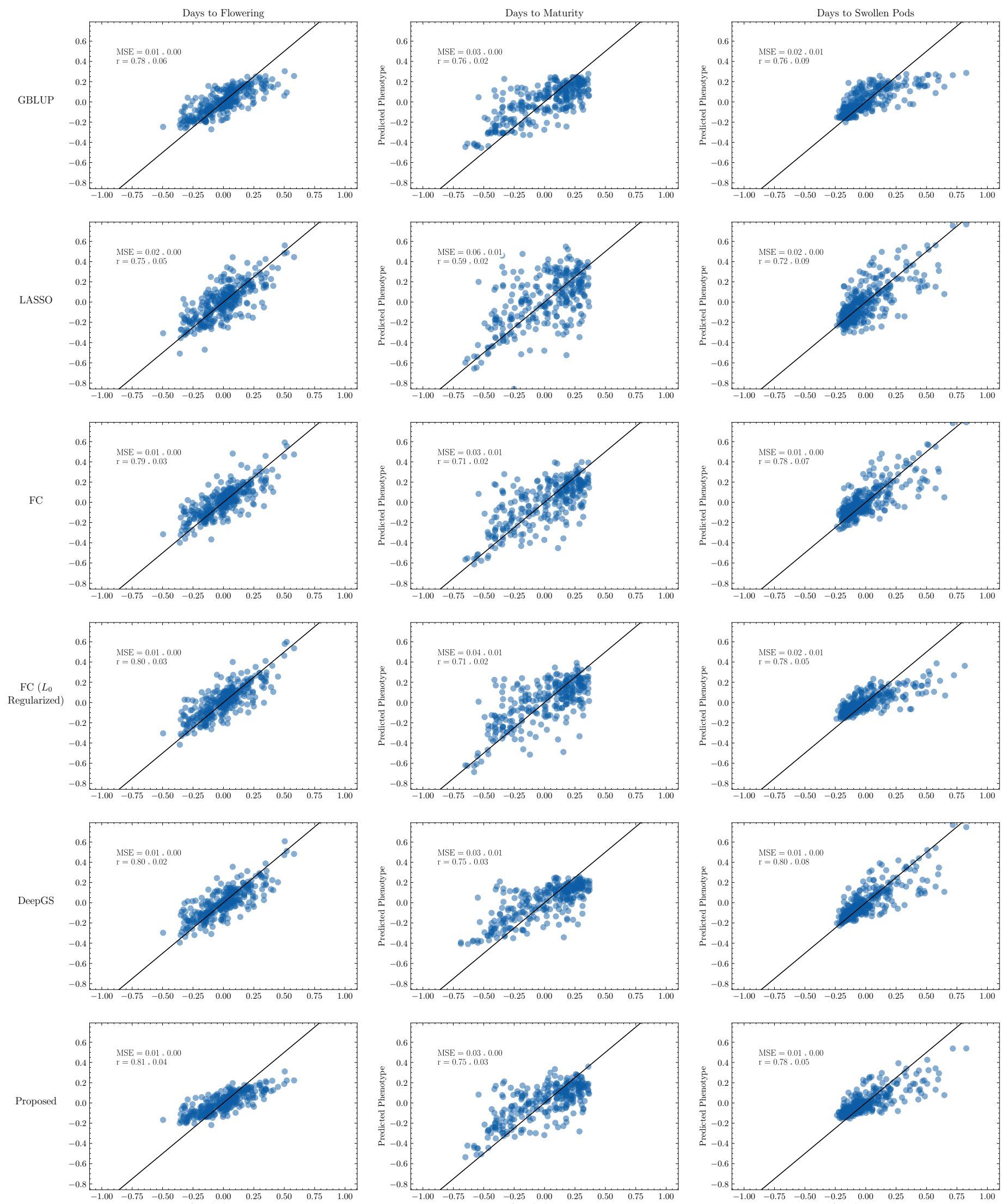

Figure S1: Detailed results for the first three traits in the lentil diversity panel dataset. The line $y = x$ is shown in black.

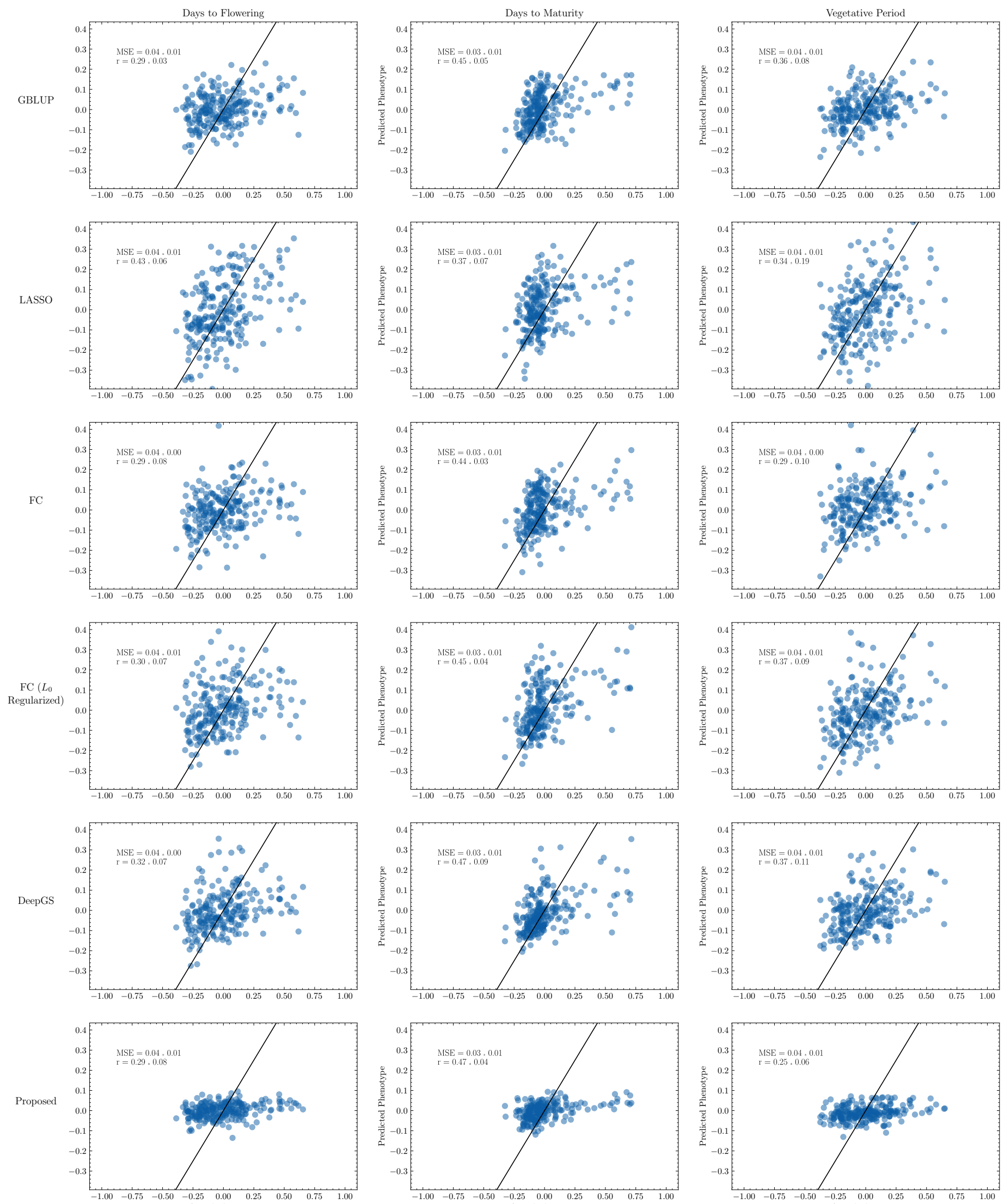

Figure S2: Detailed results for the first three traits in the lentil biparental dataset. The line $y = x$ is shown in black.

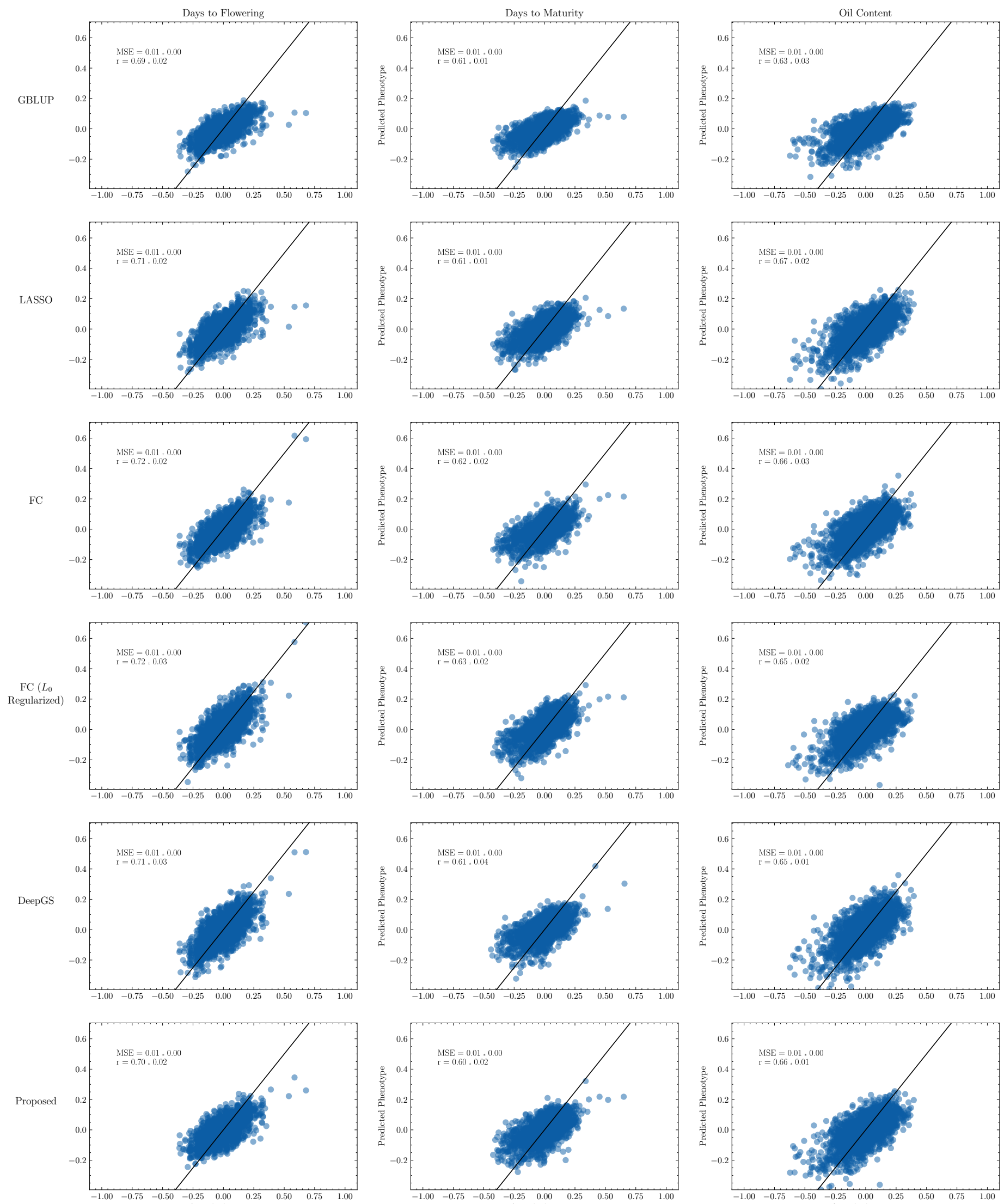

Figure S3: Detailed results for the first three traits in the carinata dataset. The line $y = x$ is shown in black.

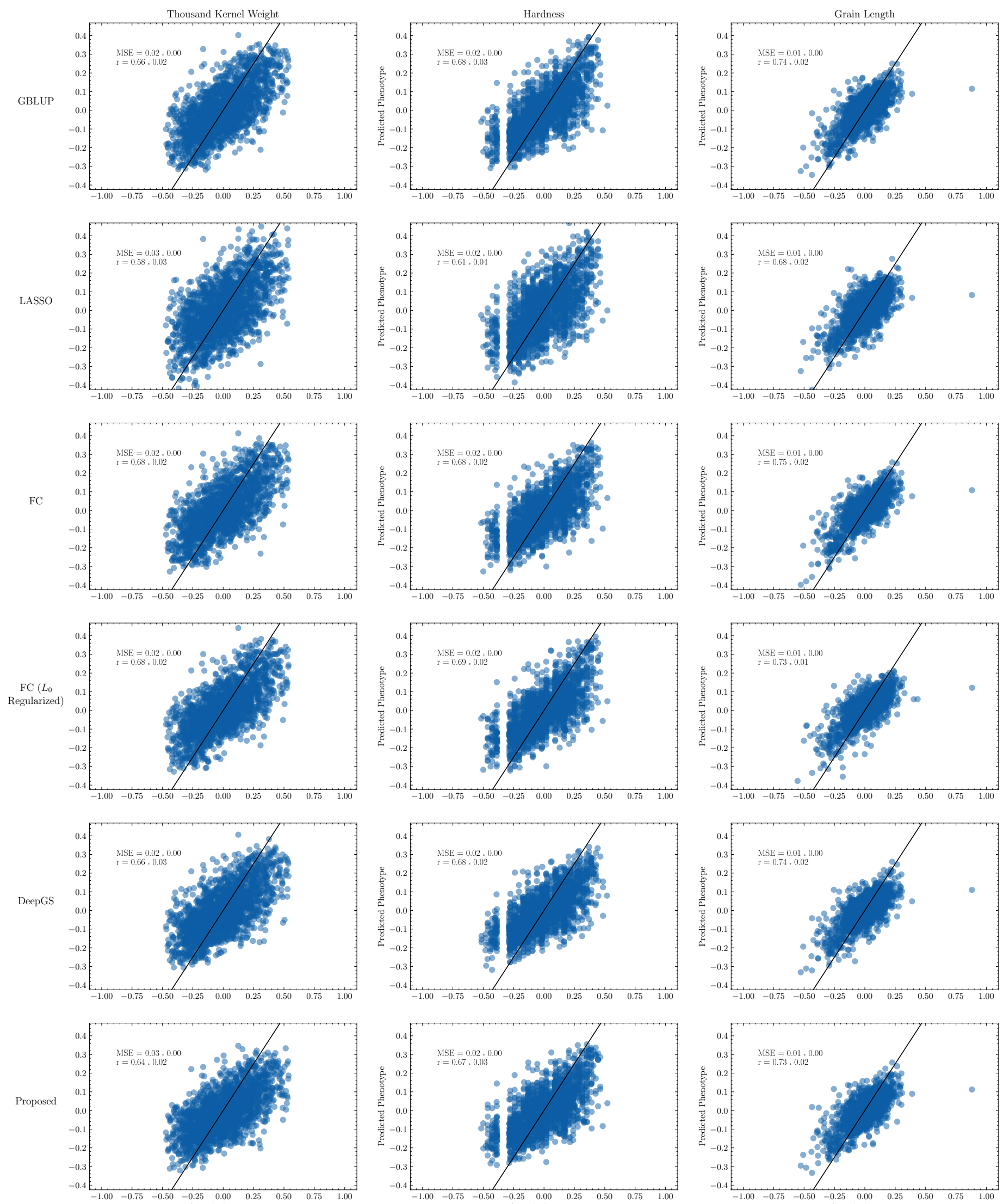

Figure S4: Detailed results for the first three traits in the wheat dataset. The line $y = x$ is shown in black.
